# GLiNER-BioMed: a suite of efficient models for open biomedical named entity recognition

**DOI:** 10.1093/bioinformatics/btag322

**Published:** 2026-05-22

**Authors:** Anthony Yazdani, Ihor Stepanov, Douglas Teodoro

**Affiliations:** Department of Radiology and Medical Informatics, Faculty of Medicine, University of Geneva, Geneva, 1202, Switzerland; Knowledgator Engineering, Kyiv, Ukraine; Department of Radiology and Medical Informatics, Faculty of Medicine, University of Geneva, Geneva, 1202, Switzerland

## Abstract

**Motivation:**

Biomedical named entity recognition (NER) presents unique challenges due to specialized vocabularies, the sheer volume of entities, and the continuous emergence of novel entities. Traditional NER models, constrained by fixed taxonomies and human annotations, struggle to generalize beyond predefined entity types.

**Results:**

To address these issues, we introduce GLiNER-BioMed, a domain-adapted suite of GLiNER models for biomedicine. Our approach first distills the annotation capabilities of large language models (LLMs) into a smaller, more efficient model, enabling the generation of high-coverage biomedical NER data. We subsequently train two GLiNER architectures, uni- and bi-encoder, at multiple scales to balance computational efficiency and performance. Experiments on eight biomedical datasets demonstrate that GLiNER-BioMed achieved state-of-the-art zero-shot performance (micro-F1 59.77%), exceeding the strongest baseline by 5.96 points (*P* < .001). In few-shot learning, the bi-encoder variant reached 70.39% (10-shot), consistently outperforming the strongest baseline across all settings (*P* < .05). Our findings show that the uni-encoder GLiNER-BioMed achieves the strongest zero-shot performance, while the bi-encoder offers superior few-shot gains and substantially higher inference throughput (+39%–568%), making it well-suited to annotation-limited, latency-sensitive, or large-label-space settings. Ablation studies further indicate that combining synthetic biomedical pre-training with general-domain post-training is essential for capturing domain-specific knowledge while maintaining precision-recall balance.

**Availability and implementation:**

The source code, datasets, and models are publicly available at https://github.com/ds4dh/GLiNER-biomed.

## 1 Introduction

**Figure 1:**
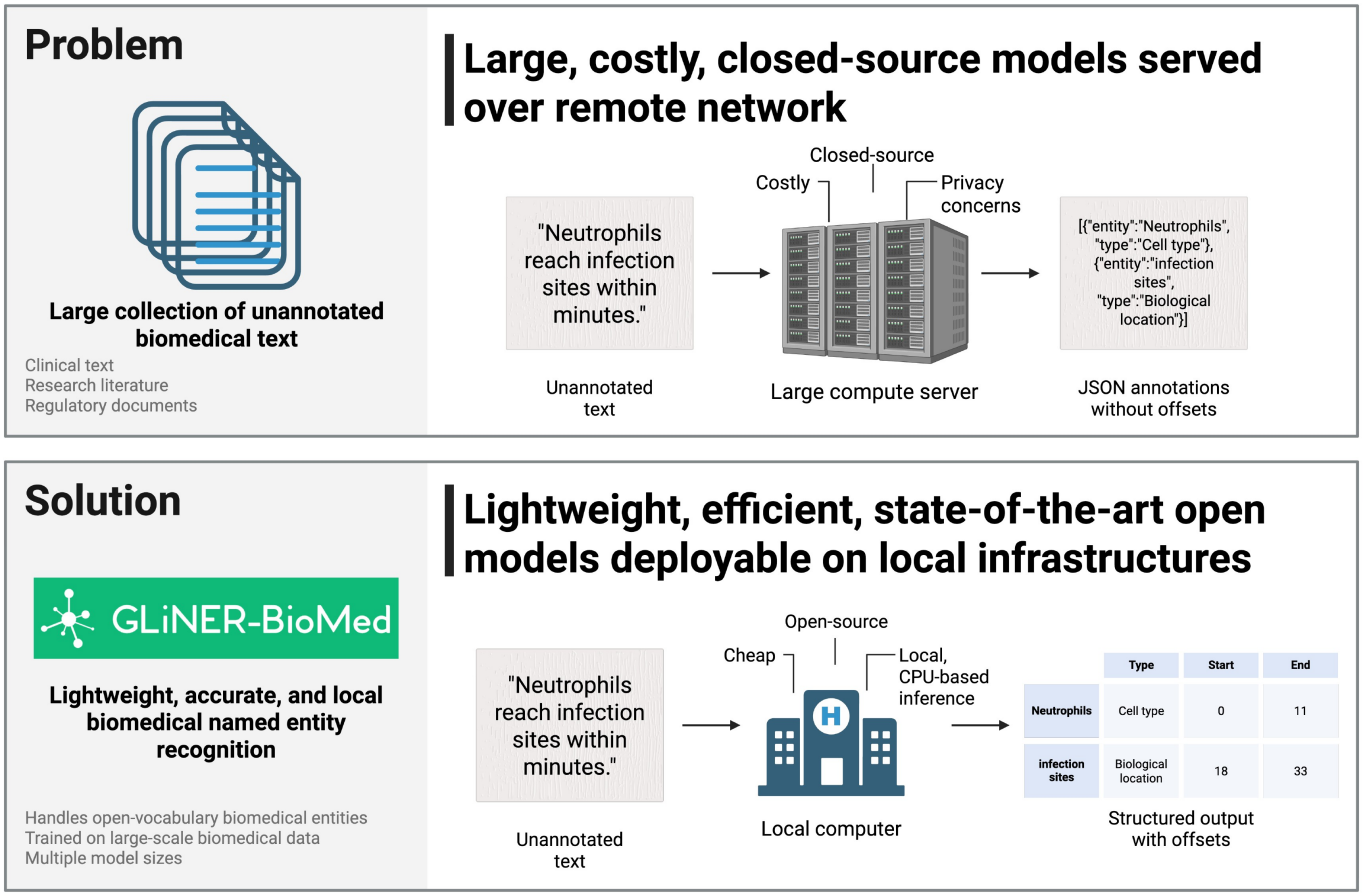
Overview of the motivation for GLiNER-BioMed. Biomedical research and clinical practice generate substantial amounts of unstructured text that require efficient methods for extracting meaningful entities. The figure contrasts traditional annotation pipelines with GLiNER-BioMed, which performs open-vocabulary biomedical NER using compact models suitable for local deployment.

Named entity recognition (NER) is a key task in biomedical natural language processing, facilitating the automated extraction of entities such as diseases, genes, and chemicals from biomedical texts (Fig. 1). As biomedical knowledge evolves, NER models must adapt to emerging terminology, diverse subdomains, and highly specialized vocabularies (Park *et al.* 2024).

Early biomedical NER systems were primarily rule-based or dictionary-driven, such as MetaMap (Aronson and Lang 2010) and cTAKES (Savova *et al.* 2010), relying on structured biomedical ontologies like UMLS (Bodenreider 2004). These approaches provided high precision for known terms but suffered from low recall and poor generalization to novel or polysemous entities (Park *et al.* 2024). Statistical methods like conditional random fields improved generalization but required extensive feature engineering and human annotations (Lafferty *et al.* 2001, Xu *et al.* 2012, Liu *et al.* 2015). The emergence of transformer-based architectures such as BioBERT (Lee *et al.* 2020), significantly advanced biomedical NER by utilizing contextual embeddings and transfer learning from domain-specific text (Lee *et al.* 2020, Yazdani *et al.* 2022, Li *et al.* 2024, Yazdani *et al.* 2025). However, the conventional NER approach using pre-trained language models, in which the classification head is fine-tuned using a fixed set of pre-defined entities, limits inference to this entity set (Devlin *et al.* 2019). As a result, models struggle to generalize beyond predefined labels, limiting their ability to recognize new, domain-specific, or emerging entities (Laparra *et al.* 2021, Jolly *et al.* 2024).

Addressing these issues, Zaratiana *et al.* (2024) introduced GLiNER, an efficient encoder-based alternative that leverages natural language label types. GLiNER’s key innovation lies in framing NER as a matching problem within a single encoder that jointly represents text and labels, enabling a lightweight and generalizable model for information extraction tasks. GLiNER consistently outperformed generative models like ChatGPT and fine-tuned GPT-style models, operating at a fraction of their parameter size and computational cost. Despite GLiNER’s promising performance in open NER, directly applying it to biomedical texts remains challenging due to specialized vocabulary, sheer volume of entities, and complex semantic structures unique to biomedical corpora (Lee *et al.* 2020, Gu *et al.* 2022). Dedicated biomedical adaptation is thus essential. To address this gap, we introduce GLiNER-BioMed, a domain-adapted suite of GLiNER models optimized for biomedical NER performance. Our contributions include:

- **Synthetic data generation:** We developed two datasets for the pre- and post-training stages of GLiNER-BioMed: (i) a synthetic biomedical pre-training dataset with 2.3 million entity mentions; and (ii) a general-domain, multi-task post-training dataset with 337 000 mentions.
- **GLiNER-BioMed models:** We pre-trained and released six GLiNER-BioMed variants across three model sizes and two architectural configurations, designed to balance zero-shot accuracy, few-shot adaptability, and inference efficiency.
- **Large-scale evaluation:** We evaluated GLiNER-BioMed models on eight biomedical NER benchmarks comprising 85 959 entity mentions across 58 entity types. In zero-shot, GLiNER-BioMed-large improves over the strongest encoder by +5.96 F1 and over the best generative model by +11.22 F1. In few-shot, GLiNER-BioMed-bi-large reaches 70.39% with only 10-shot supervision, consistently outperforming the strongest baseline.
- **Open-source release:** We publicly release all resources developed in this work, including our pre- and post-training corpora, six GLiNER-BioMed models, and one distilled synthetic-annotation model. The source code, datasets, and models are publicly available at https://github.com/ds4dh/GLiNER-biomed.

Recent open NER approaches, also known as universal or generalist NER, have moved beyond fixed taxonomies by reframing entity recognition as reading comprehension or prompt-based tasks. For example, Li *et al.* (2020) reformulated NER as question answering, while Aly *et al.* (2021) used type descriptions to classify entities from unseen classes. Similarly, Cocchieri *et al.* (2025) proposed OpenBioNER, a BERT-based cross-encoder that injects entity descriptions into token classification to improve generalization to novel biomedical entities. Though significantly smaller, OpenBioNER showed a marginal performance increase over GLiNER-v1.0-large, the first released version of the model.

Parallel efforts have explored generative models for zero- and few-shot NER. The unified information extraction (UIE) framework (Lu *et al.* 2022) unified entity, relation, event, and sentiment extraction into a single text-to-structure generation task. Zhou et al. (2024) distilled ChatGPT-generated annotations into UniversalNER (UniNER), achieving strong performance in open NER. In the biomedical domain, Keloth *et al.* (2024) instruction-tuned LLaMA-7B to create BioNER-LLaMA, which matches the performance of specialized NER models. Recently, PP-UIE (https://github.com/PaddlePaddle/PaddleNLP/releases/tag/v3.0.0-beta4) extended UIE using the Qwen2 family of foundation models (Yang*et al.* 2025) for multi-task extraction over longer contexts. Despite these advances, generative approaches continue to face challenges related to computational cost and inference latency (Dietrich and Hollstein 2025).

The GLiNER framework itself has also seen continued development. Subsequent iterations (v2.0, v2.1, v2.5) aimed to improve zero-shot performance. Variants or domain-adapted versions have also been proposed, such as NuNER-Zero, which incorporates annotations generated by large language models (LLMs) for enhanced token and span recognition (Bogdanov *et al.* 2024), and GLiNER-news, optimized specifically for the news domain (Törnquist and Caulk 2024). However, a dedicated biomedical adaptation remained lacking. GLiNER-BioMed addresses this by extending the GLiNER framework for biomedical NER through large-scale synthetic pre-training on biomedical data.

## 2 Materials and methods

To create GLiNER-BioMed, we first constructed a large-scale synthetic pre-training dataset tailored to biomedical NER. To further enhance zero-shot generalization, we then leveraged a post-training dataset from the general domain. Finally, we investigated two architectural variants of GLiNER-BioMed, with different computational complexities, across multiple model scales, each of which underwent pre- and post-training stages.

### 2.1 Pre-training dataset

**Figure 2:**
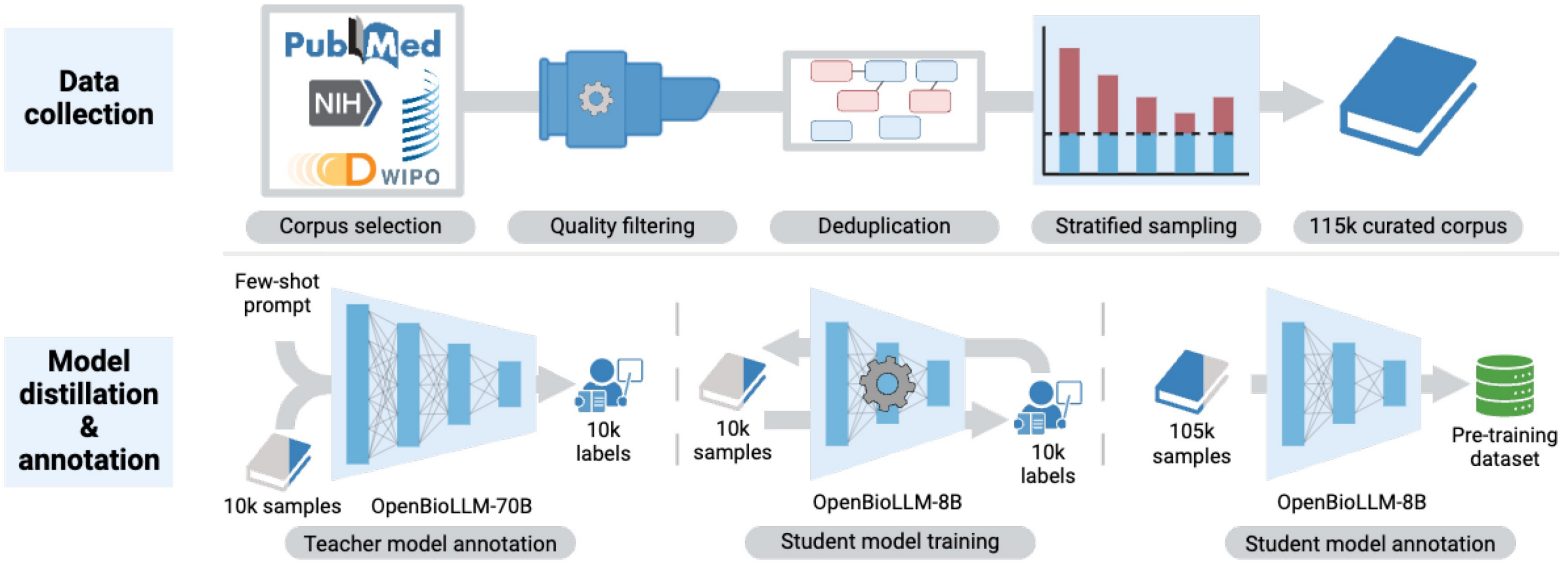
Overview of the synthetic pre-training data generation pipeline for GLiNER-BioMed. The pipeline begins with data collection, involving corpus selection, quality filtering, deduplication, and stratified sampling to produce a 115k passage corpus. This curated corpus is then annotated using a model distillation strategy where OpenBioLLM-70B (teacher) annotates an initial 10k samples, which are used to train a smaller OpenBioLLM-8B (student) model using low-rank adaptation. The distilled student model then efficiently annotates the remaining 105k passages for the final pre-training dataset.

To develop a high-coverage biomedical NER dataset, we first assembled a corpus that encompasses a broad spectrum of biomedical knowledge, including scientific literature (PubMed, https://pubmed.ncbi.nlm.nih.gov), clinical trials (ClinicalTrials.gov, https://clinicaltrials.gov), human prescription labels (DailyMed, https://dailymed.nlm.nih.gov/dailymed), as well as biomedical patents (WIPO, https://ipcpub.wipo.int). This diverse set of unlabeled biomedical corpora would later be synthetically annotated using a generative LLM. Below, we describe our methodology for corpus selection, quality filtering, deduplication, and synthetic annotation. The complete pipeline is depicted in Fig. 2.

#### 2.1.1 Corpus selection

PubMed abstracts indexed between 1 January and 16 December 2024, under the MeSH term pathological conditions, signs, and symptoms were collected using the NCBI Entrez Programming Utilities (https://www.ncbi.nlm.nih.gov/books/NBK25501/). This MeSH term was selected as a pragmatic filter to focus on human medicine and clinical presentations. For clinical trials, we extracted detailed study descriptions as well as arm-level treatment regimens (i.e. descriptions of the specific interventions assigned to each arm) from all trials registered up to 28 November 2024. From DailyMed, we retrieved all available human prescription label sections. Finally, biomedical patent descriptions registered with the WIPO were sourced under the categories A61P (Specific therapeutic activity of chemical compounds or medicinal preparations), G16H (Healthcare informatics), and A61K (Preparations for medical, dental or toiletry purposes) of the International Patent Classification.

#### 2.1.2 Quality filtering

We implemented a heuristic-based pipeline to filter low-quality passages. A passage is defined here as any unit of text, such as an abstract or a section, treated as a single input sample. Text quality was quantified using a combination of lexical and structural metrics, and passages falling below predefined thresholds were excluded. We applied tailored, more stringent criteria for clinical trial treatment regimen descriptions due to their structural specificity.

Specifically, for human prescription labels, clinical trial detailed descriptions, PubMed abstracts, and biomedical patents, we required passages to contain no more than 30% words with non-alphabetic characters, at least 6 sentences, and an average sentence length of 10 words or more. The proportion of uppercase letters among all alphabetic characters was limited to a maximum of 20%, and no single word could exceed 20% of the total word count to avoid excessive repetition. Passages were also removed if they contained too many line breaks, defined as more than one newline cluster for every two sentences. Additionally, we ensured at least 10% lexical diversity, meaning at least 10% of the words needed to be unique, and required a minimum of 5% common English stopwords. Due to their distinctive formatting, we applied more stringent criteria for filtering clinical trial treatment regimens. Passages were retained only if they began with a capital letter, included at least one digit, ended with a period, and contained at least two well-formed sentences.

#### 2.1.3 Deduplication and content diversity

**Figure 3:**
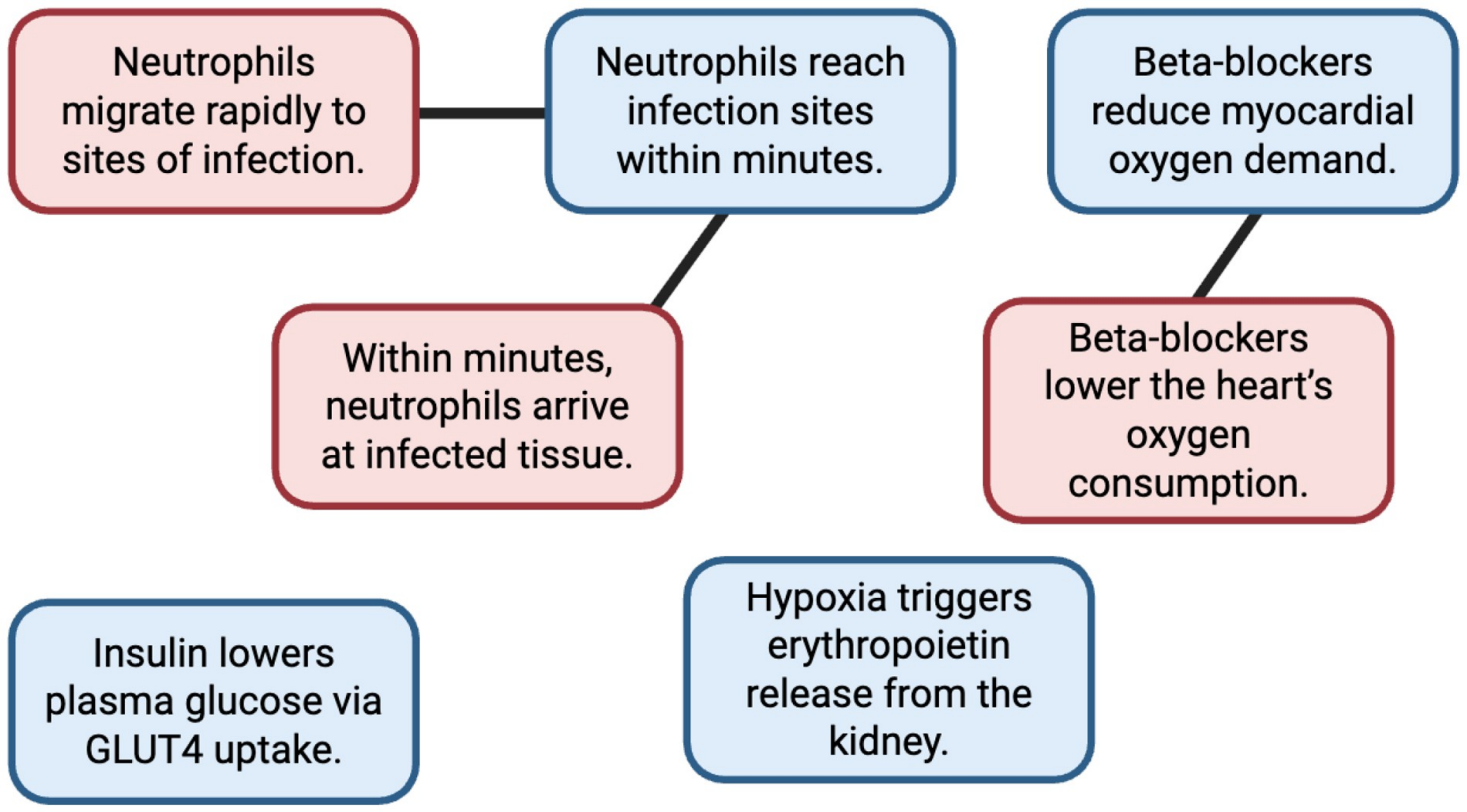
TF-IDF similarity graph for biomedical passages. Blue nodes represent retained representatives; red nodes indicate excluded duplicates.

To mitigate redundancy, we applied a graph-based deduplication strategy to each biomedical corpus independently. Text passages were first transformed into TF-IDF representations. We then constructed a similarity graph, where nodes represented individual texts and edges were formed between passages exceeding a cosine similarity of 0.9. Within this graph, connected components emerged as clusters of redundant texts. To retain a maximally informative yet diverse corpus, the passage with the highest average similarity to others was selected as the canonical representative, and all the other instances were excluded. This process is depicted in Fig. 3. Detailed evaluations of cluster quality and chaining effects for each corpus are provided in Supplementary Information 1, available as supplementary data at *Bioinformatics* online.

#### 2.1.4 Stratified sampling

Next, we performed stratified sampling to construct a balanced dataset for synthetic NER annotation. Prior to sampling, our curated corpus comprised 106 982 PubMed abstracts, 76 145 clinical trial descriptions, 31 778 prescription label sections, 88 867 arm-level treatment regimens, and 114 609 patent descriptions. To optimize computational efficiency while preserving domain diversity, we randomly selected approximately 115 000 passages with equal representation from all sources.

#### 2.1.5 Model distillation and annotation

To construct a large-scale, synthetically annotated NER dataset, we designed a multi-stage process integrating few-shot prompting and model distillation. First, from the balanced subset obtained in the previous step, 10 000 samples were used to create high-quality NER annotations using the OpenBioLLM-70B (https://huggingface.co/aaditya/Llama3-OpenBioLLM-70B) model. In a few-shot setting, incorporating four in-context examples, OpenBioLLM-70B was prompted to annotate these initial 10 000 samples. The four in-context examples were manually annotated from passages in our curated corpus. A brief excerpt of the prompt is provided in Supplementary Information 2, available as supplementary data at *Bioinformatics* online, and the full prompt is provided in our codebase.

To control the scope of annotation, we extracted noun phrases from the input using spaCy (Montani *et al.* 2023) and provided them to the model as candidate entities. The model was then prompted to classify each noun phrase according to its entity type. This constraint ensured that potentially relevant entities were not omitted. To facilitate parsing, guided decoding was applied to enforce JSON-formatted outputs (Kwon *et al.* 2023).

Subsequently, we fine-tuned a student model, OpenBioLLM-8B, using low-rank adaptation (Hu *et al.* 2022) to internalize the open-vocabulary noun-phrase annotation behavior of its larger counterpart using these 10 000 annotated samples. This distillation eliminated the need for in-context examples during inference, significantly reducing context length requirements and making annotation more efficient. The student model was then used to generate annotations for the remaining 105 000 samples.

#### 2.1.6 Synthetic pre-training dataset

**Figure 4:**
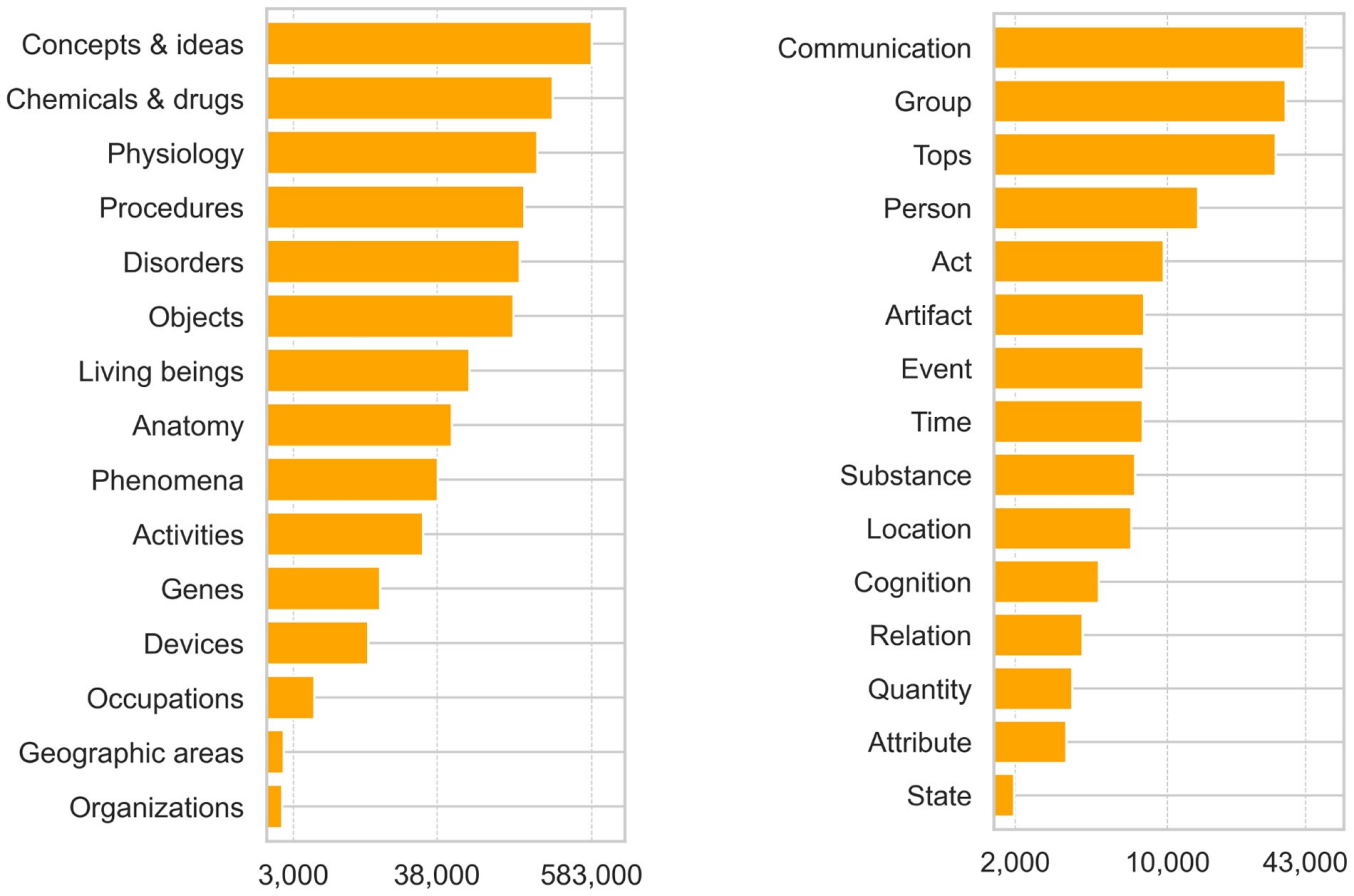
Left. UMLS semantic groups by mention count (log scale) in the biomedical synthetic pre-training dataset. Counts reflect total NER label-to-semantic group mappings; labels associated with multiple semantic groups contribute to multiple bars. Right. Top 15 WordNet lexnames by mention count (log scale) in the post-training dataset. Each mention is assigned a single lexname based on its most frequent WordNet sense.

The synthetic pre-training dataset comprises 105 000 samples, 2.3 million entity mentions covering 640 000 unique entities. For exploratory data analysis, 1.5 million of these labels were successfully linked to UMLS concepts via exact string matching, yielding over 2 million CUIs distributed across 120 of the 127 UMLS semantic types and all 15 UMLS semantic groups. This indicates broad coverage of biomedical concepts, encompassing the vast majority of semantic types and all defined semantic groups. Figure 4 (left) presents the distribution of synthetic biomedical NER labels across UMLS semantic groups.

### 2.2 Post-training dataset

To enhance zero-shot performance, we build upon and expand the dataset introduced by Stepanov and Shtopko (2024) as the foundation for our post-training data. The base dataset consists of 5000 instances, combining manually curated examples with synthetic multi-task annotations. Specifically, it includes 1878 manually curated examples drawn from WNUT2017 (Derczynski *et al.* 2017), OntoNotes5 (Hovy *et al.* 2006), and MultiNERD (Tedeschi and Navigli 2022). In addition, it contains 3122 synthetic examples derived from English Wikipedia articles. These synthetic examples were annotated using Llama-3-8B (Grattafiori *et al.* 2024), prompted to perform multiple information extraction (IE) tasks, including NER, open IE, and relation extraction, all formulated as entity recognition tasks to align with the GLiNER framework.

In this work, we substantially augmented this base dataset by generating 14 000 additional synthetic examples, following the same methodology. For this augmentation phase, we used the FineWeb dataset (Penedo *et al.* 2024) as the source and annotated the text using Qwen2.5-72B (Yang*et al.* 2025) . Altogether, the final post-training dataset comprises 19 000 instances, with 337 000 annotated mentions spanning 12 700 unique labels. To assess the semantic breadth of the NER labels, we mapped entity labels to WordNet, finding that 195 000 labels matched WordNet entries across 37 of all 45 lexnames, and 26 out of 26 noun lexnames. A breakdown of the 15 most frequent lexnames is presented in Fig. 4 (right).

### 2.3 Model architectures and training

GLiNER-BioMed uses two distinct model architectures, uni- and bi-encoder, with varied computational complexities. Each architecture was trained across three parameter scales (small, base, and large).

#### 2.3.1 Uni-encoder architecture

The standard uni-encoder GLiNER architecture (Zaratiana *et al.* 2024) uses a single transformer encoder $f_{\text{enc}}$ to jointly process text and entity types. Given a tokenized input text passage *T* and a set of *k* target entity types $E={\left\{e_{i}\right\}}_{i=1}^{k}$, an input sequence $X=[S_{E},T]$ is constructed, where $S_{E}$ is a tokenized representation of *E*, constructed by formatting the entity types with special tokens. The encoder processes this combined sequence, generating contextualized hidden states:


$$H=f_{\text{enc}}(X).$$

**Figure 5:**
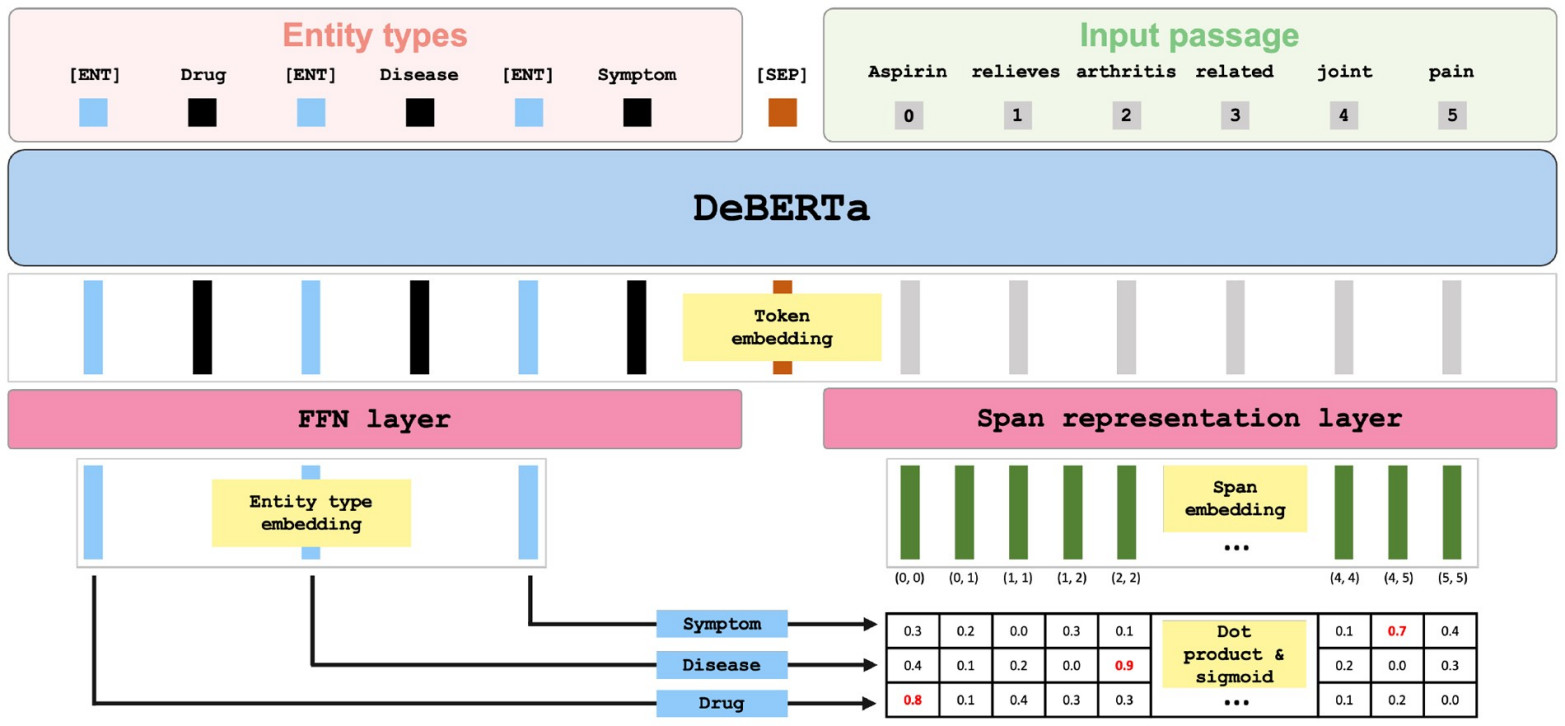
Uni-encoder GLiNER-BioMed architecture. This architecture concatenates entity types with the input passage into a single sequence, allowing the model to learn cross-dependencies between the text and the candidate labels before computing span classification scores.

From the resulting contextualized hidden states *H*, the text token representations $H_{T}$ are extracted. Similarly, a set of *k* distinct vector representations for the entity types, $H_{E}={\left\{h_{i}\right\}}_{i=1}^{k}$, is also derived from *H*. These representations are subsequently used to compute span-to-entity-type scores. We evaluate all possible spans up to a maximum length of 12 words. This architecture is depicted in Fig. 5.

For our implementation, we use DeBERTa-v3 (He *et al.* 2023) as the encoder component $f_{\text{enc}}$. Specifically, GLiNER-BioMed-small, -base, and -large are built on the small (141M parameter), base (184M), and large (434M) variants of DeBERTa-v3, respectively. We adopt DeBERTa-v3 to maintain direct comparability with general-domain GLiNER models.

#### 2.3.2 Bi-encoder architecture

GLiNER’s computational complexity is dominated by the self-attention mechanism over the combined input, which scales as $O({\left(|S_{E}|+|T|\right)}^{2})$. This becomes prohibitive in settings with a large number of candidate entity types. The bi-encoder model proposed in this work leverages a recently introduced variant of GLiNER (Stepanov *et al.* 2026). This architecture uses two distinct encoders, one for the input text passage, $f_{\text{enc}}^{T}$, and one for entity types, $f_{\text{enc}}^{E}$. The text *T* and entity types *E* are processed as follows:


$$H_{T}=f_{\text{enc}}^{T}(T),\;H_{E}={\left\{f_{\text{enc}}^{E}(e_{i})\right\}}_{i=1}^{k}.$$

**Figure 6:**
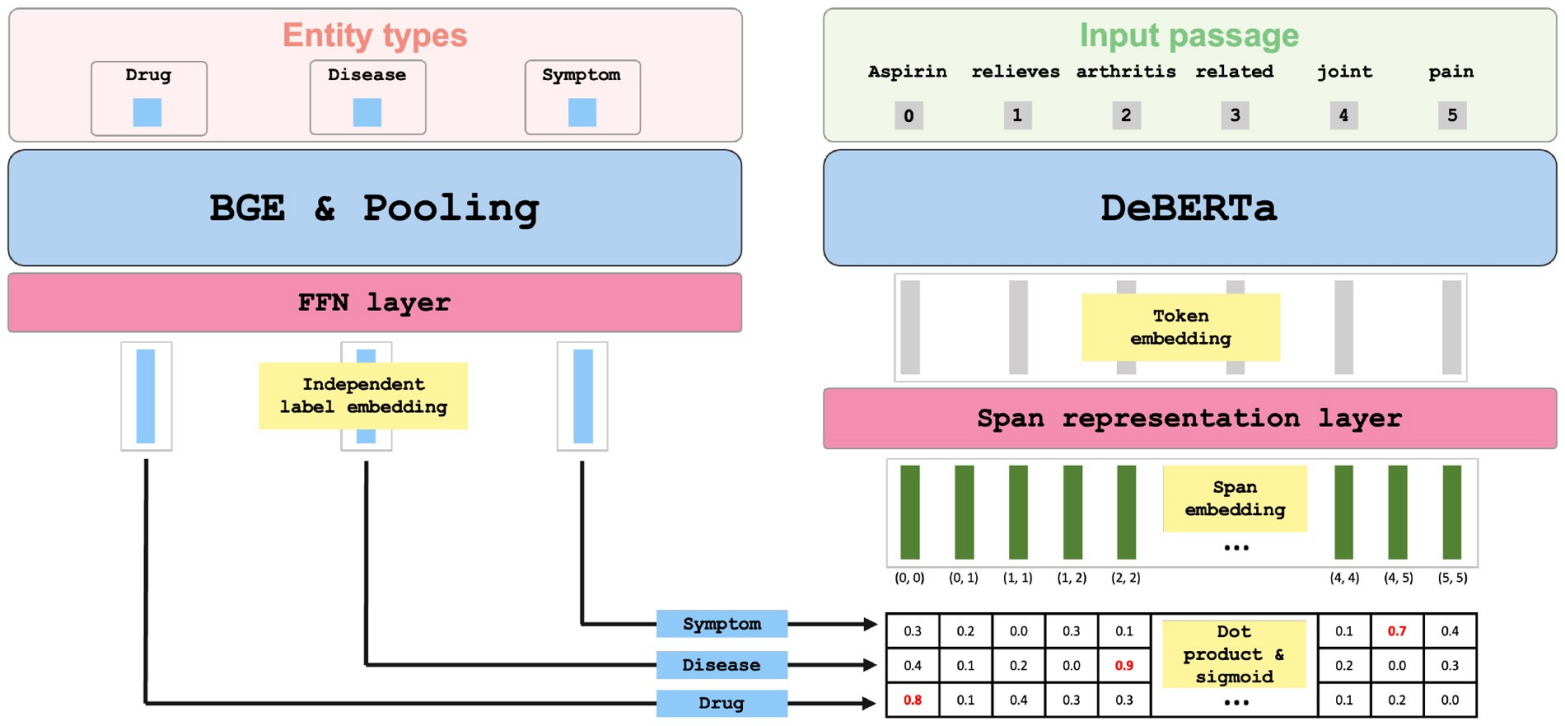
Bi-encoder GLiNER-BioMed architecture. This architecture decouples the encoding of entity types and the input passage, allowing label representations to be pre-computed and cached for efficient inference.

The label representations $H_{E}$ are computed independently of the text and of one another. As a result, they can be pre-computed and cached, offering significant efficiency gains in scenarios involving a large number of entity types. The span-to-entity-type scoring procedure remains the same as in the uni-encoder setup. This separation results in a more favorable complexity profile, with online computation scaling primarily with text length, $O(|T{|}^{2})$, and a smaller, offline cost for encoding the entity types. Consistent with the uni-encoder architecture, we apply the same maximum span length of 12 words. The architecture is shown in Fig. 6.

For our implementation, which we refer to as GLiNER-BioMed-bi-small, -base, and -large, we adopt DeBERTa-v3 (small/base/large) as the text encoder backbones $f_{\text{enc}}^{T}$, following the uni-encoder setup. For the label encoders $f_{\text{enc}}^{E}$, we use all-MiniLM-L6-v2 (Reimers and Gurevych 2019) (23M parameters) for small, BGE-small-en-v1.5 (Xiao *et al.* 2024) (33M) for base, and BGE-base-en-v1.5 (Xiao *et al.* 2024) (109M) for large.

#### 2.3.3 Training procedure

All model variants underwent pre- and post-training stages. Uni-encoder models were pre-trained exclusively on our fully synthetic biomedical dataset. Bi-encoder models, which have a larger parameter count and thus require broader data exposure to converge, were pre-trained on both the NuNER corpus (Bogdanov *et al.* 2024) and our synthetic biomedical dataset. In a second stage, all models were fine-tuned on the post-training dataset, allowing for broader coverage of general-domain entity types and linguistic contexts.

The pre- and post-training datasets were split into 90% training and 10% validation sets. During synthetic biomedical pre-training, models were trained for 20 000 steps with a batch size of 8, using the AdamW optimizer (Loshchilov and Hutter 2019) with a weight decay of 0.01. The learning rate was set to $1\times 10^{-5}$ for the encoder backbones and $5\times 10^{-5}$ for all other model parameters. For post-training, models were trained for 10 000 steps with a batch size of 4, again using AdamW with a weight decay of 0.01. The learning rate during this phase was reduced to $5\times 10^{-6}$ for the encoder backbones and $1\times 10^{-5}$ for the remaining parameters.

## 3 Results

### 3.1 Evaluation

We evaluate the performance of GLiNER-BioMed models in both zero-shot and few-shot settings. Unless stated otherwise, we use three lenient performance metrics (i.e. a prediction is considered a true positive if the predicted entity type matches the ground truth type and the predicted span overlaps with the ground truth span): (i) micro F1-score, which provides an overall measure of precision-recall balance; (ii) macro mean F1-score, which averages F1-scores per entity type, treating all entity categories equally regardless of their frequency; and (iii) macro median F1-score, which reports the median F1-score across all entity types, mitigating the influence of outliers. To enable comparison with generative models, which do not natively produce span offsets, we locate predicted entities by matching generated entity strings to the input passage.

For statistical comparisons, we assess the differences between two models using a Wilcoxon signed-rank test on their per-passage F1-scores, specifically testing whether Model A’s F1-scores exceed Model B’s. A low *P*-value provides significant evidence that Model A outperforms Model B; a high *P*-value implies there is insufficient evidence for that claim. Finally, we evaluate GLiNER-BioMed models in a fully fine-tuned setting, directly comparing their performance with supervised state-of-the-art results reported in the literature using exact span matching.

Beyond accuracy, we examine the practical impact of computational complexity in GLiNER model variants, comparing how uni- and bi-encoder architectures differ in inference throughput when handling varying numbers of entity types.

#### 3.1.1 Benchmark datasets

For the evaluation, we consider eight publicly available, human-annotated NER benchmarks covering a broad range of biomedical subdomains, including clinical narratives, scientific abstracts, regulatory documents, drug labels, and patient-generated content. These include TAC (Roberts *et al.* 2017), CADEC (Karimi *et al.* 2015), N2C2 2018 (Henry *et al.* 2020), BC5CDR (Li *et al.* 2016), BioRED (Luo *et al.* 2022), CHIA (Kury *et al.* 2020), Biomed NER (https://huggingface.co/datasets/knowledgator/biomed_NER), and NCBI Disease (Doğan *et al.* 2014). In total, these datasets comprise 10 918 text passages and 85 959 entity mentions spanning 58 unique biomedical entity types. Supplementary Information 3, available as supplementary data at *Bioinformatics* online details the dataset descriptions, label nomenclature, and preprocessing steps, alongside coverage statistics for the model’s 12-word maximum span length threshold.

#### 3.1.2 Results on zero-shot performance

We evaluate GLiNER-BioMed-large models in zero-shot settings against a range of GLiNER and generative baselines, as shown in Table 1. Corresponding zero-shot results for the small and base models are presented in Supplementary Information 4, available as supplementary data at *Bioinformatics* online. The GLiNER baselines include the general-purpose versions v1.0 (Zaratiana *et al.* 2024), v2.0, v2.1, and v2.5, as well as NuNER-Zero, NuNER-Zero-span (Bogdanov *et al.* 2024), and GLiNER-news-v2.1 (Törnquist and Caulk 2024). The generative baselines include biomedical chat models, namely, OpenBioLLM in 8B and 70B; general-domain chat models, Qwen3 in 8B and 32B (Yang *et al.* 2025); and five dedicated information extraction models, including UniNER-7B (Zhou et al. 2024) and PP-UIE in 0.5B, 1.5B, 7B, and 14B configurations.

**Table 1:** Zero-shot NER performance of GLiNER-BioMed-large models against seven GLiNER-large baselines and nine generative LLMs, aggregated over eight datasets.^a^

Model	Micro F1	Macro mean F1	Macro median F1
**Generative LLMs**			
OpenBioLLM-70B^b^	36.30	20.86	14.40
Qwen3-32B^c^	37.67	26.55	22.27
PP-UIE-14B	25.74	18.05	13.73
OpenBioLLM-8B	15.95	9.29	3.95
Qwen3-8B^c^	34.04	21.47	10.84
UniNER-7B	48.55	30.35	28.10
PP-UIE-7B	24.73	17.30	13.25
PP-UIE-1.5B	23.87	16.30	11.59
PP-UIE-0.5B	19.78	13.27	9.27
**GLiNER-large**			
NuNER-Zero	40.87	21.79	13.94
NuNER-Zero-span	40.26	22.51	14.27
GLiNER-v1.0	47.77	29.60	21.13
GLiNER-v2.0	37.38	21.42	15.44
GLiNER-v2.1	48.04	29.75	28.20
GLiNER-news-v2.1	48.99	31.79	33.77
GLiNER-v2.5	53.81	35.22	35.65
GLiNER-BioMed^d^	**59.77**	**40.67**	**42.65**
GLiNER-BioMed-bi^e^	54.90	35.78	31.66

a Bold: best; underlined: second-best.

b Q4KM-quantized.

c Thinking mode disabled.

d 478-million-parameter model.

e 569-million-parameter model.

As shown in Table 1, GLiNER-BioMed-large outperforms all baselines in zero-shot settings. Against other GLiNER-large models, it achieves a micro F1-score of 59.77%, improving by 5.96 points over the next-best model, GLiNER-v2.5-large (*P* < .001). Against generative LLMs, GLiNER-BioMed-large also delivers stronger zero-shot performance. It surpasses UniNER-7B by 11.22 points (*P* < .001) and outperforms the top-performing PP-UIE model by a wide margin (*P* < .001). OpenBioLLM-70B and OpenBioLLM-8B reach F1-scores of 36.30% and 15.95%, respectively. These results show that GLiNER-BioMed not only surpasses state-of-the-art baselines but also the foundation models used to annotate its training data (*P* < .001 in both cases). The GLiNER-BioMed-bi-large model also exhibits strong performance; however, it remains 4.87 points lower than that of the uni-encoder (*P* < .001).

Table 2 reports micro-averaged F1-scores for GLiNER-BioMed-large, GLiNER-BioMed-bi-large, and GLiNER-v2.5-large on each of the eight evaluation datasets. Overall, at least one of the two GLiNER-BioMed variants outperforms GLiNER-v2.5 on all datasets, with particularly pronounced gains on BioRED (+10.2 points), CADEC (+8.27), N2C2 (+5.81), and TAC (+17.07), indicating substantial benefits from biomedical specialization. GLiNER-BioMed-large achieves the highest F1 on five of eight datasets (Biomed NER, BioRED, CADEC, N2C2, TAC), while the bi-encoder performs best on the remaining three (BC5CDR, CHIA, NCBI Disease).

**Table 2:** Dataset-level, zero-shot micro-averaged F1-scores of GLiNER-BioMed-large models against GLiNER-v2.5-large.^a^

Dataset	BioMed-bi	BioMed	v2.5
BC5CDR	**82.02**	77.09	80.80
Biomed NER	46.19	**47.19**	42.71
BioRED	71.01	**74.90**	64.70
CADEC	25.44	**34.42**	26.15
CHIA	**46.84**	46.57	43.45
N2C2	51.41	**60.49**	54.68
NCBI disease	**77.40**	64.68	74.25
TAC	51.75	**64.40**	47.33

a Bold: best; underlined: second-best.

#### 3.1.3 Results on fine-tuned performance

We evaluate GLiNER-BioMed-large models in the fully supervised setting. The results are compared with dataset-level state-of-the-art (SOTA) systems, GLiNER-v2.5-large and a PubMedBERT (Gu *et al.* 2022) model trained for NER using HuggingFace’s implementation (Wolf *et al.* 2020) (PMB), as summarized in Table 3.

**Table 3:** Fully supervised NER performance of GLiNER-BioMed-large and GLiNER-BioMed-bi-large compared with state-of-the-art systems, GLiNER-v2.5-large, and PMB for each dataset.^a^

Dataset	BioMed-bi	BioMed	v2.5	PMB	SOTA
BC5CDR	88.12	87.76	87.52	88.73	**91.96** (Wang *et al.* 2024)
Biomed NER^b^	51.39	**52.61**	51.86	51.12	51.86 (Zaratiana *et al.* 2024)
BioRED	90.49	89.71	88.13	89.53	**91.26** (Luo *et al.* 2023)
CADEC^c^	75.97	**77.71**	76.99	71.43	75.69 (Huang *et al.* 2023)
CHIA	**65.95**	64.72	64.99	50.93	65.78 (Tian *et al.* 2021)
N2C2	**90.11**	90.02	89.85	88.40	89.56 (Henry *et al.* 2020)
NCBI Disease	86.18	85.61	85.34	85.61	**91.10** (Panahi 2025)
TAC^c^	88.29	89.16	**89.48**	87.50	82.48 (Roberts *et al.* 2017)

a Scores are exact span micro F1-scores. Bold: best per dataset; underlined: second-best.

b For Biomed NER, SOTA is a fine-tuned GLiNER-v2.5-large baseline because no publicly available SOTA result could be identified.

c Discontinuous entities were disregarded.

Under full supervision, GLiNER-BioMed variants set a new SOTA in 4 out of 8 datasets. On N2C2, both uni- and bi-encoder variants achieve scores above SOTA, with the bi-encoder reaching 90.11. Strong performance is also observed on CADEC and TAC, where both models exceed SOTA, although discontinuous entities were disregarded from the datasets, making direct comparison less straightforward. On CHIA and Biomed NER, results are mixed but remain within one point of SOTA, with the bi-encoder slightly stronger on CHIA and the uni-encoder on Biomed NER.

By contrast, performance drops are observed on BC5CDR and NCBI Disease, where GLiNER-BioMed, GLiNER-v2.5-large, and PMB models fall short of SOTA by several points. NCBI Disease consists exclusively of disease mentions, while BC5CDR covers both diseases and chemicals. A closer analysis shows that this underperformance stems specifically from the disease entity type. On BC5CDR, the uni-encoder achieves an F1 of 83.11 and the bi-encoder 83.51 on disease mentions, while both exceed 91 on chemicals. This difficulty is not unique to GLiNER-BioMed, as the same trend is observed in GLiNER-v2.5-large and PMB. This suggests that the challenge lies in the complexity of disease mentions within these benchmarks rather than a model-specific deficiency. Across datasets, the two GLiNER-BioMed architectures remain very close in absolute terms, with differences typically under one point.

To better understand model behavior, we selected and organized fine-grained entity labels across datasets into a set of clinically relevant groups that capture higher-level semantic categories commonly used in biomedical text. “Disease,” “Disorder,” “Condition,” and “Reason for drug prescription” were merged into a unified “Disease/Diagnosis” group, while “Drug administration route,” “Dosage,” “Strength,” “Frequency of administration,” “Treatment duration,” “Drug form,” and “Drug class” were combined under “Regimen attribute” to represent medication-specific properties. Similarly, “Chemical” and “Signaling molecule” were grouped as “Chemical,” and medical procedures formed the “Procedure” group. Other categories, such as “Drug,” “Adverse drug event” (ADE), “Gene/Protein,” and “Variant,” were retained as single-label groups. Table 4 presents the resulting F1-scores, highlighting the strengths and weaknesses of GLiNER-BioMed large models across these categories.

**Table 4:** Fine-tuned NER performance of GLiNER-BioMed-large models versus GLiNER-v2.5-large and PMB aggregated by clinically relevant entity groups.^a^

Entity group	BioMed-bi	BioMed	v2.5	PMB
ADE	91.37	91.66	**91.72**	90.43
Chemical	86.33	86.32	85.19	**87.94**
Disease/Diagnosis	**84.23**	83.31	83.72	82.42
Drug	**93.62**	93.37	93.00	92.49
Gene/Protein	78.85	78.74	78.25	**82.65**
Procedure	**63.73**	62.90	63.16	59.64
Regimen attribute	**95.45**	95.35	95.14	95.16
Variant	**92.02**	91.03	87.20	89.59

a Bold: best; underlined: second-best. ADE: Adverse drug event.

#### 3.1.4 Results on few-shot performance

Few-shot learning is particularly valuable in biomedical contexts, where annotated data is often limited due to the high cost and domain expertise required for manual labeling. To assess model performance under such constrained supervision, we fine-tune GLiNER-BioMed-large and GLiNER-BioMed-bi-large on 10-, 20-, and 50-shot subsets drawn from the training splits of each dataset, and compare them to GLiNER-v2.5-large and PMB. Identical subsets were used across all models. These were constructed by prioritizing passages that ensure full coverage of all entity types, filling the remaining capacity with examples containing the highest number of unique entity types. All models are evaluated on full test sets for consistency with zero-shot results.

**Figure 7:**
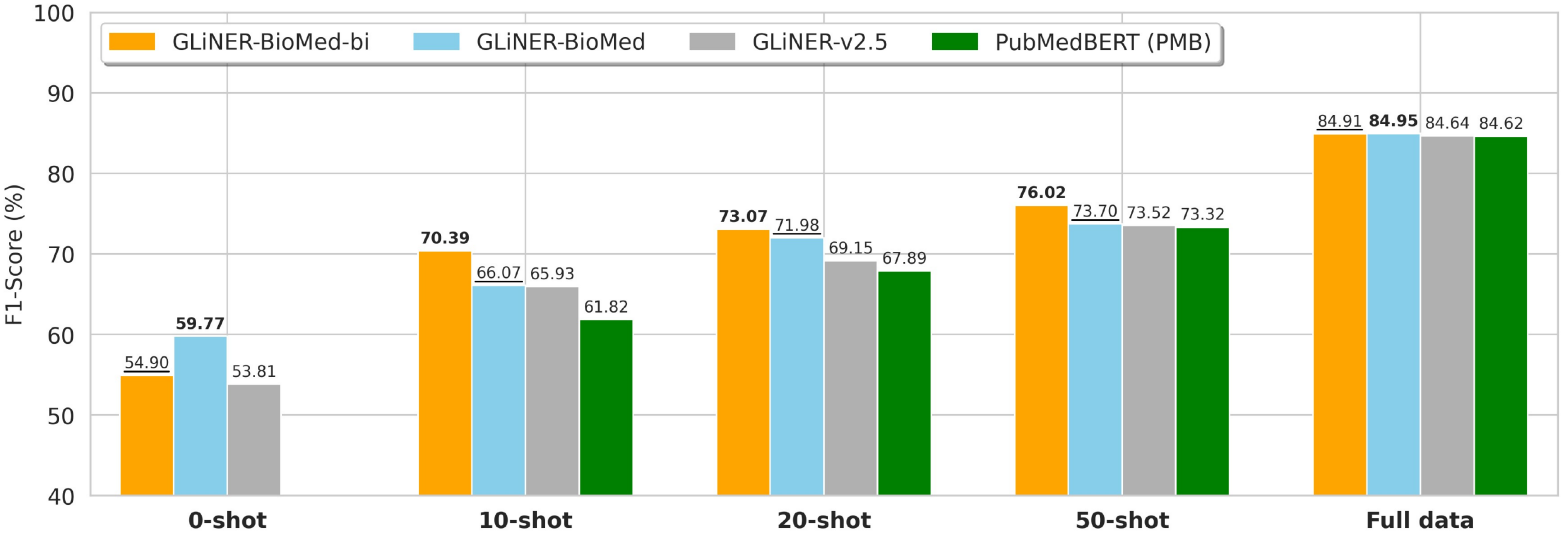
Few-shot NER performance of GLiNER-BioMed large models versus GLiNER-v2.5-large and PMB, aggregated over eight datasets using micro F1-scores. N-shot denotes N training and validation samples. Bold: best per setting; underlined: second-best. PMB cannot perform zero-shot NER.

As shown in Fig. 7, GLiNER-BioMed-bi-large consistently outperforms GLiNER-v2.5-large and uni-encoder versions in the few-shot setting, reaching a 70.39% micro F1-score with only 10 examples, representing a 4.46 point improvement over GLiNER-v2.5-large (*P* < .001). This advantage over GLiNER-v2.5-large holds at 20 and 50-shot, with *P* < .05 and *P* < .001, respectively. The uni-encoder GLiNER-BioMed-large also performs well in these N-shot scenarios, slightly outperforming GLiNER-v2.5-large, although not statistically significantly (*P* > .05 for all comparisons). Under full supervision, all models converge. In this setting, both GLiNER-BioMed variants perform comparably to GLiNER-v2.5-large, with *P* > .05 for all comparisons. This result suggests that the uni-encoder GLiNER-BioMed is best suited for zero-shot scenarios, while the bi-encoder variant excels in annotation-constrained settings, improving performance upon zero-shot as much as 15.49 points (*P* < .001), with only 10 training samples.

#### 3.1.5 Uni- versus bi-encoder inference efficiency

The practical utility of NER models critically depends on inference efficiency. To evaluate this, we benchmarked the large GLiNER-BioMed and GLiNER-BioMed-bi variants on an NVIDIA RTX3090 GPU, measuring peak throughput in words per second using FP32 precision. Evaluations were conducted under two conditions: (i) using the original set of entity types for each dataset, and (ii) using a set comprising all 127 UMLS semantic types.

**Figure 8:**
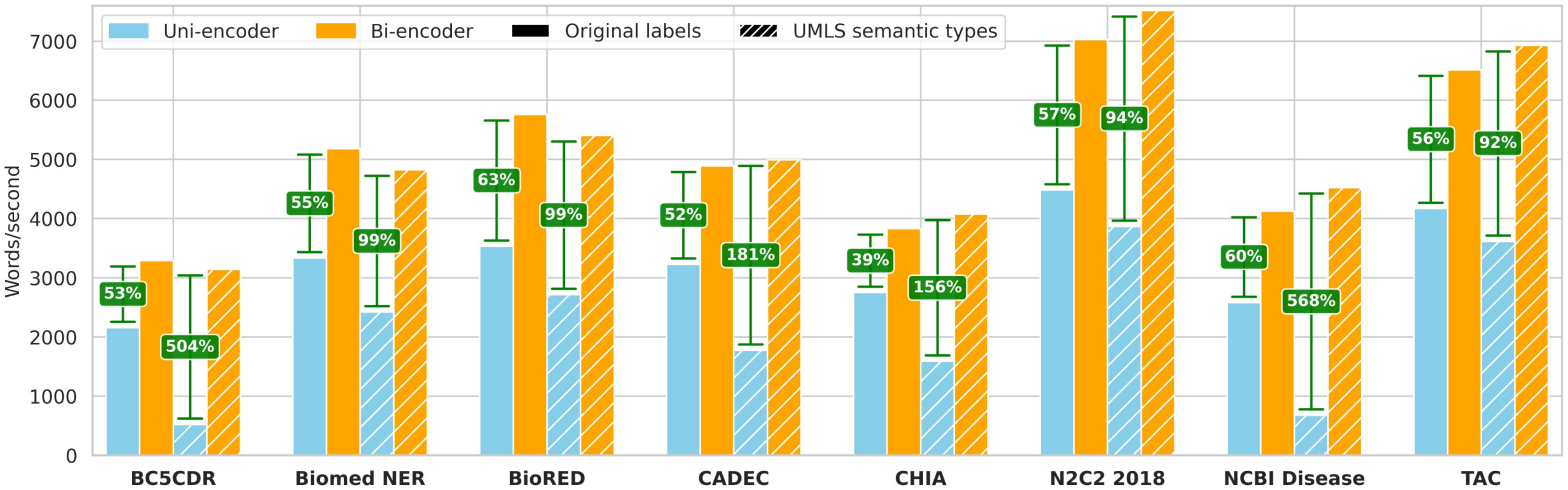
Peak inference throughput (words/second) for GLiNER-BioMed large models, illustrating architectural efficiency under varied entity type loads. Peak words/second is the maximum achieved over batch sizes going from 1 to 64 on an NVIDIA RTX3090 GPU before out-of-memory using FP32 precision. Performance of uni-encoder (blue) and bi-encoder (orange) is compared using (i) dataset-specific entity labels (solid bars), and (ii) a fixed set of 127 UMLS semantic types (hatched bars). Annotated percentages indicate the throughput difference between bi- and uni-encoders.

As shown in Fig. 8, the bi-encoder outperformed the uni-encoder in throughput in all settings. With dataset-specific labels, it was between 39% and 63% faster. This performance gap widened substantially under the full UMLS label set, where the uni-encoder processes text and labels jointly through self-attention, causing inference time to grow quadratically with the combined sequence length. In contrast, the bi-encoder decouples text and label encoding, allowing label embeddings to be pre-computed and cached, drastically reducing inference overhead. Under the 127-label setting, this resulted in speedups ranging from 92% to 568%, depending on the dataset.

### 3.2 Analysis of the teacher model: LLMs are strong entity typers but weak span detectors

A core premise of GLiNER-BioMed is that LLMs can serve as effective teachers for generating synthetic training data. While OpenBioLLM-70B achieves a modest micro F1-score of 36.30% in a zero-shot NER setting, this metric conflates two distinct capabilities: (i) span detection (localizing the entity) and (ii) entity typing (assigning an entity type to a span). To motivate the design of our data synthesis pipeline, which initially tasks OpenBioLLM-70B with typing pre-extracted noun phrases, we designed an experiment to decouple and measure these capabilities using the eight benchmark datasets. In this experiment, we provided the model with both the text passage and the gold-standard spans and prompted it to perform entity typing alone.

As shown in Table 5, entity typing is a task where the LLM strongly outperforms its NER performance. Across the eight benchmarks, the aggregated micro F1-score increases from 36.30% (NER) to 78.59% (entity typing). These results indicate that LLMs experience difficulty with the joint task of span detection and entity typing (i.e. NER), rather than lacking the semantic knowledge necessary for entity typing alone.

**Table 5:** Zero-shot F1-scores comparing OpenBioLLM-70B performance in NER versus entity typing across the eight benchmark datasets.

Dataset	NER	Entity typing	Δ
BC5CDR	68.34	98.59	+30.25
Biomed NER	24.05	54.69	+30.64
BioRED	49.94	96.1	+46.16
CADEC	26.44	32.64	+6.2
CHIA	32.25	64.59	+32.34
N2C2	24.24	81.8	+57.56
NCBI Disease	57.99	100.00	+42.01
TAC	41.89	93.81	+51.92
Aggregated	36.30	78.59	+42.29

This evaluation imposes a strict closed-set constraint, forcing the model to choose from the dataset labels. A confusion analysis of the lower-performing datasets reveals that most errors stem from semantic overlaps. In CADEC, the low F1-score of 32.64% is driven almost entirely by the model classifying adverse drug events as symptoms, a specific disagreement that accounts for 86.2% of all errors. In Biomed NER, errors are driven by high granularity, with the most frequent misclassifications being “Function” versus “Activity” (7.7% of errors) and “Phenotype” versus “Finding” (7.3%). In the CHIA dataset, the model most frequently misclassifies “Scope” as “Condition” (11.4% of errors) or “Value” as “Measurement” (6.9%). From a synthetic annotation perspective, the LLM’s tendency toward hypernymy might be acceptable. For example, while a specific term like “Adverse drug event” might be best suited for a given dataset’s schema, assigning an umbrella term like “Symptom” remains semantically correct and a valid training signal.

### 3.3 Ablation studies

We perform ablation studies to quantify the synergies of synthetic biomedical pre-training and general-domain post-training on GLiNER-large models.

As shown in Table 6, GLiNER-v2.5-large, trained solely on general-domain data, achieves 53.81% micro F1-score. Further training GLiNER-v2.5-large on our post-training data yields a modest gain to 54.80%, suggesting some benefit from high-quality annotations but limited domain adaptation. Training a randomly initialized GLiNER model on our post-training data alone results in a similar F1-score of 52.38%, confirming the quality of the data while highlighting the need for domain-specific exposure. Pre-training a randomly initialized GLiNER model exclusively on synthetic biomedical data yields 42.10% F1-score, with high precision (70.08%) but low recall (30.09%). This result indicates that synthetic biomedical pre-training effectively imparts domain-specific knowledge but lacks sufficient recall to achieve the highest F1-score. The best performance comes from combining biomedical synthetic pre-training with general-domain post-training on a randomly initialized GLiNER checkpoint, resulting in 59.77% F1-score with balanced precision (56.67%) and recall (63.22%). Applying the same pre- and post-training pipeline to the GLiNER-v2.5-large checkpoint yields an F1-score of 58.13%. This indicates that synthetic biomedical pre-training imparts domain knowledge regardless of initialization, and that pre-existing general-domain GLiNER weights might not always offer an advantage over random initialization.

**Table 6:** Ablation study evaluating the impact of different training phases on biomedical NER performance.^a^

Initial checkpoint (GLiNER-large)	General-domain pre-training	Synthetic biomedical pre-training	General-domain post-training	Precision	Recall	F1-score
GLiNER-v2.5	**✓**	×	×	56.19	51.62	53.81
GLiNER-v2.5	**✓**	×	✓	55.56	54.06	54.80
GLiNER-v2.5	**✓**	✓	✓	60.04	56.34	58.13
Random	×	×	✓	51.53	53.25	52.38
Random	×	✓	×	**70.08**	30.09	42.10
Random	×	✓	✓	56.67	**63.22**	**59.77**

a The evaluated model configurations are: (i) GLiNER-v2.5-large, a model trained solely on general-domain data; (ii) GLiNER-v2.5-large further trained on our post-training data; (iii) GLiNER-v2.5-large further trained on our synthetic biomedical data and subsequently on our post-training data; (iv) randomly initialized GLiNER-large trained only on our post-training data; (v) randomly initialized GLiNER-large trained exclusively on our synthetic biomedical data; and (vi) GLiNER-BioMed-large, a randomly initialized GLiNER-large model trained on our synthetic biomedical data and subsequently trained on our post-training data. All reported metrics are micro-averaged. Bold: best; underlined: second-best.

Overall, the best performance is uniquely achieved by combining pre- and post-training stages. Despite leading to an overly confident model on its own, synthetic biomedical pre-training creates the necessary synergy with post-training to maximize overall recognition performance.

### 3.4 Qualitative analysis

We conducted a brief qualitative analysis to illustrate the type of entity recognition behavior exhibited by GLiNER-BioMed-large in zero-shot settings. Ten passages were manually designed to test distinct properties desirable in biomedical NER systems: (i) Compositional extraction: the ability to identify and jointly extract complementary components. (ii) Word sense disambiguation: distinguishing between semantic interpretations of the same lexical item. (iii) Negation handling: detecting explicit cues that indicate the absence of findings. (iv) Abbreviation resolution: recognizing that full expressions and their abbreviations refer to the same underlying concept. (v) Hierarchical disambiguation: distinguishing between related concepts that exist at different levels of specificity.

**Figure 9:**
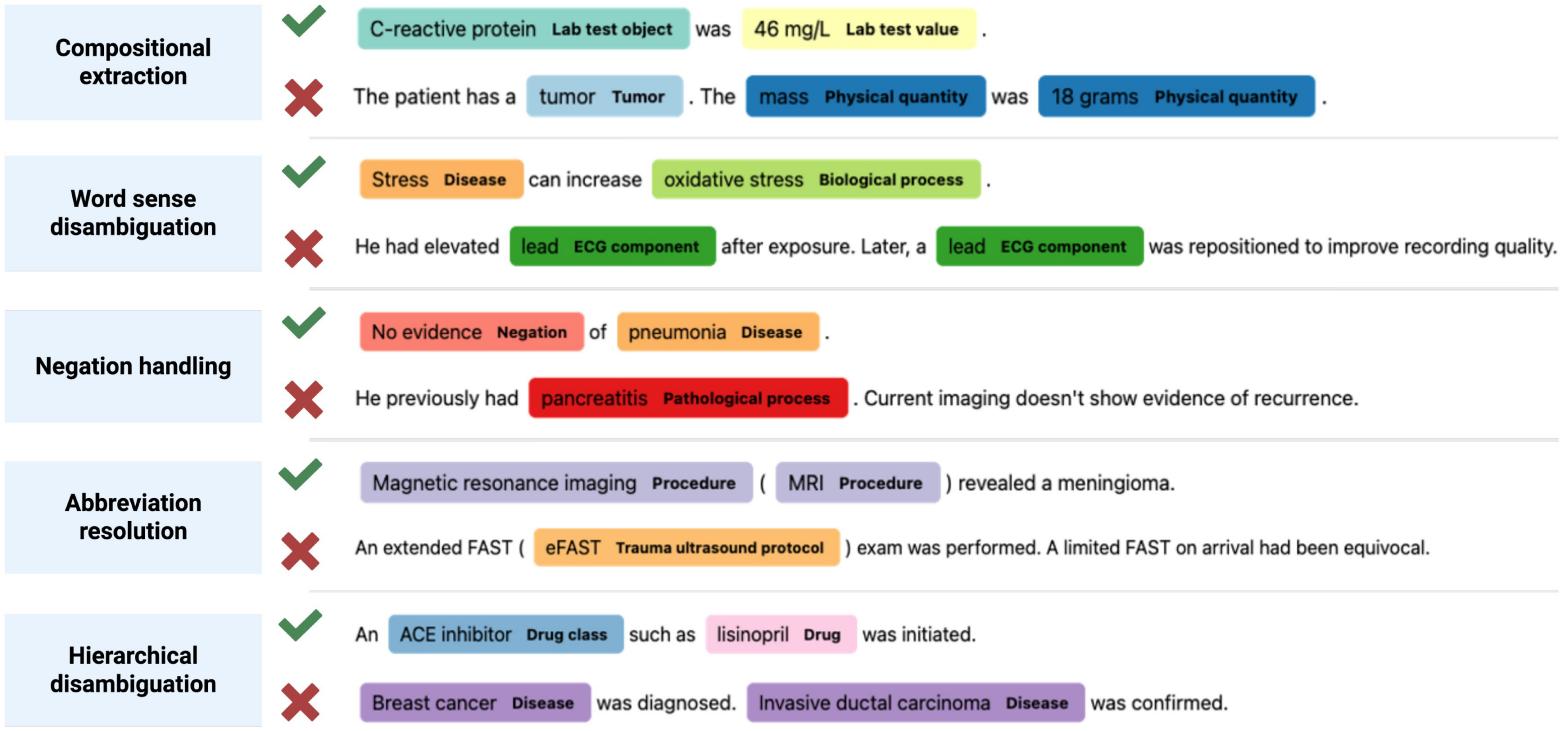
Illustrative examples of GLiNER-BioMed-large in zero-shot biomedical NER. Each row demonstrates a distinct property: (i) Compositional extraction; (ii) Word sense disambiguation; (iii) Negation handling; (iv) Abbreviation resolution; and (v) Hierarchical disambiguation.

Figure 9 illustrates each of these aspects with one successful and one failure example. In the compositional extraction case, the model correctly identifies both the laboratory test “C-reactive protein” and its associated measurement “46 mg/L,” showing its ability to capture semantically linked entities. In contrast, in a sentence describing a “tumor” followed by “The mass was 18 grams.”, the model incorrectly interprets “mass” as a physical quantity rather than another mention of the tumor itself. For word sense disambiguation, the model successfully differentiates the general clinical term “stress” from “oxidative stress,” a biological process term. In another example, it assigns the ECG-related sense of “lead” to both mentions, including the one that should be interpreted as a toxin. For negation handling, the model correctly recognizes “No evidence” as a negation cue while separately identifying “pneumonia” as a disease, whereas in a sentence describing a past episode of “pancreatitis,” it only marks the pathological process and fails to capture the explicit negation of recurrence. In the abbreviation resolution examples, the model labels both “Magnetic resonance imaging” and its shorthand “MRI” as procedures, yet for the trauma ultrasound protocol, it recognizes “eFAST” but does not mark the other abbreviation and the long form. Finally, for hierarchical disambiguation, the model appropriately distinguishes “ACE inhibitor” as a drug class and “lisinopril” as an individual drug, but in an example involving “Breast cancer” and its subtype “Invasive ductal carcinoma,” it assigns both mentions the same disease label, collapsing the distinction between a disease group and a more specific disease entity.

## 4 Discussion

In this work, we introduced GLiNER-BioMed, a suite of open NER models adapted to the biomedical domain. Unlike conventional approaches that rely on fixed taxonomies, GLiNER-BioMed uses natural language labels, enabling more flexible biomedical entity recognition. The results show that a compact encoder can match or even surpass the performance of much larger models. A key finding is that GLiNER-BioMed outperforms the foundation models used to create its training data. Our analysis of OpenBioLLM-70B indicates that while it accurately types entities, it struggles with NER itself (i.e. the joint task of span detection and entity typing). Our pipeline decouples these challenges by initially using OpenBioLLM-70B as an entity typer for pre-extracted noun phrases, leveraging its typing accuracy while bypassing its detection weaknesses. This illustrates a broader pattern emerging in information extraction, where large LLMs can serve as effective offline teachers, while smaller, task-focused models become more efficient students. This trend aligns with findings from GLiNER and UniNER, both of which demonstrate that smaller models can surpass the performance of their teachers.

The synthetic biomedical corpus plays a central role in GLiNER-BioMed’s performance. Although created without human annotation, it provides a scale that would be prohibitively expensive to obtain manually. Its coverage across nearly all UMLS semantic types exposes the model to a wide variety of biomedical entities. When used in isolation, however, it pushes the model toward high precision but low recall, resulting in an overly cautious system. General-domain post-training corrects this imbalance, and the best performance is achieved only when both stages are combined. This sequential improvement indicates positive transfer rather than catastrophic forgetting, likely due to the shared training objective. However, the lower performance of biomedical-only training compared to general-domain-only training suggests that the synthetic biomedical data can be further improved. Future work could explore using larger, more capable LLMs for annotation.

The architectural comparison shows that the two GLiNER-BioMed variants meet different practical needs. The uni-encoder achieves the strongest zero-shot performance, while the bi-encoder overtakes it as soon as small amounts of supervision are available. We hypothesize that the uni-encoder is best in zero-shot settings because its cross-attention mechanism allows to contextualize label representations as a function of the input passage and other candidate labels. Conversely, in few-shot scenarios, the absence of this cross-attention in the bi-encoder might act as a regularizer, preventing the overfitting that can occur in low-data regimes. Interestingly, in zero-shot settings, the bi-encoder outperforms the uni-encoder specifically on BC5CDR and NCBI Disease, datasets with only two and one entity types, respectively. We suspect the uni-encoder might be sensitive to the number of labels at inference time, especially in scenarios where the number of entity types is low compared to its training data. However, these hypotheses require further exploration to be confirmed. The bi-encoder, by contrast, is insensitive to the number of entity types by construction. Additionally, the ability to pre-compute label embeddings gives the bi-encoder a clear inference-speed advantage, particularly when the label space is large. When fine-tuned, both models perform consistently well on drug-related categories such as doses, regimens, and adverse drug events. In contrast, performance on diseases, procedures, and gene-related mentions is lower. The qualitative examples illustrate different ways in which the models can fail, including failing to capture compositional entities, missing negation mentions, handling abbreviations unevenly, collapsing distinctions between general and specific entity variants, and misinterpreting terms that carry multiple meanings. These errors are not unique to GLiNER-BioMed and reflect broader challenges in biomedical NER where contextual cues and semantic granularity can be difficult to resolve, even with substantial domain adaptation.

Although GLiNER-BioMed achieves substantial gains, several limitations remain. First, our synthetic pre-training data, generated via distilled generative models, may introduce biases or fail to fully capture the complexity inherent in human-annotated data, potentially limiting generalizability. Additionally, despite their diversity, the evaluation datasets may not represent all biomedical subdomains or linguistic variations, especially less common areas, such as veterinary medicine or dentistry. While GLiNER-BioMed’s high inference throughput makes it suitable for processing full-text articles, the scientific literature portion of its training data consisted exclusively of abstracts. As noted by Cohen *et al.* (2010), full-text bodies differ substantially from abstracts, and performance on such documents remains to be verified. In addition, although GLiNER-BioMed is designed to overcome the constraints of fixed taxonomies, our evaluation focused on established benchmarks, and its ability to generalize to arbitrary entity types remains to be systematically characterized. The computational requirements of the full pipeline may also limit reproducibility and accessibility for researchers in resource-constrained environments. Finally, our use of DeBERTa-v3 was motivated by comparability with general-domain GLiNER baselines, but this choice may limit performance in biomedical settings. Future work could explore biomedical-specific backbones or alternative GLiNER checkpoint initializations.

## 5 Conclusion

This work introduces GLiNER-BioMed, a specialized suite of open biomedical NER models. Unlike conventional approaches that rely on fixed taxonomies, GLiNER-BioMed incorporates natural language labels, enabling more flexible and adaptive biomedical entity recognition. Our approach begins by distilling annotations from a large generative model into a smaller generative model, which is then used to generate high-coverage synthetic biomedical NER data. We then pre-train GLiNER models on this synthetic dataset, followed by post-training on general-domain data, enhancing annotation accuracy while preserving strong domain adaptation. Through extensive zero-shot and few-shot evaluations across eight biomedical datasets, GLiNER-BioMed consistently outperforms state-of-the-art information extraction models, achieving a 5.96-point F1-score improvement over the strongest baseline. Additionally, the bi-encoder variant proves particularly effective in low-data settings, achieving a 70.39% micro F1-score with as few as 10 training samples. Beyond accuracy, GLiNER-BioMed-bi offers substantial efficiency gains, achieving up to 568% higher throughput compared to the uni-encoder. Future research could explore more capable and scalable generative large language models for synthetic data annotations, multilingual adaptations, alternative encoder backbones, and continual learning strategies to ensure robust adaptation to the ever-evolving biomedical landscape.

## Supplementary Material

btag322_Supplementary_Data

## Data Availability

The pre-training dataset is publicly available on Hugging Face at https://huggingface.co/datasets/anthonyyazdaniml/gliner-biomed-pre-training. The post-training dataset is publicly available on Hugging Face at https://huggingface.co/datasets/anthonyyazdaniml/gliner-biomed-post-training. All GLiNER-BioMed models, along with the synthetic annotation model, can be accessed at https://github.com/ds4dh/GLiNER-biomed (DOI: 10.5281/zenodo.18710501).
