# Supplementary material for: GLiNER-BioMed: a suite of efficient models for open biomedical named entity recognition
Source: Bioinformatics. 2026 May 22;42(6):btag322. doi: 10.1093/bioinformatics/btag322 (PMC13259603; doi:10.1093/bioinformatics/btag322)
Supplement: btag322_Supplementary_Data [file btag322_supplementary_data.pdf]

GLiNER-BioMed: A Suite of Efficient Models for Open Biomedical Named Entity Recognition

Anthony Yazdani[1,*], Ihor Stepanov[2], Douglas Teodoro[1]

[1] Department of Radiology and Medical Informatics, University of Geneva, Geneva, Switzerland

[2] Knowledgator Engineering, Kyiv, Ukraine

[*] Corresponding author

- E-mail address: anthony.yazdani@unige.ch

Keywords: Named entity recognition, NER, Biomedical, Zero-shot, Few-shot

SUPPLEMENTARY INFORMATION

S1. Deduplication cluster quality analysis

To evaluate the quality of the resulting clusters and quantify chaining effects in our deduplication method, where less similar texts might merge via intermediate nodes in the graph, we calculated the minimum intra-cluster similarity (cluster purity). A cluster is considered to demonstrate a chaining effect if the minimum pairwise similarity between any two distinct nodes within it falls below the similarity threshold (0.9). For PubMed abstracts, the process reduced 107 024 passages to 106 982 clusters (0.04% reduction), averaging 1.00 passage per cluster, with an average purity of 0.999993 and no detected chaining effects. Clinical trial descriptions produced 76 145 clusters from 77 244 inputs (1.42% reduction), averaging 1.01 passages per cluster, with an average purity of 0.999392 and chaining in only 0.03% of clusters. Treatment regimens resulted in 88 867 clusters from 104 330 inputs (14.82% reduction), averaging 1.17 passages per cluster, with an average purity of 0.994183 and chaining in 0.79% of clusters. Similarly, patent descriptions yielded 114 609 clusters

from 141 849 inputs (19.20% reduction), averaging 1.24 passages per cluster, with an average purity of 0.995969 and chaining in 0.57% of clusters. Lastly, prescription labels showed the highest redundancy, condensing 138 018 sections into 31 778 clusters (a 76.98% reduction), averaging 4.34 passages per cluster. This subset contained the largest clusters and the highest chaining rate (7.8%), reflecting the standardized nature of regulatory text, yet it still achieved an average purity of 0.975085.

S2. Teacher model prompt

```
<|begin_of_text|><|start_header_id|>system<|end_header_id|>

You are an advanced assistant trained to process biomedical text for
Named Entity Recognition (NER) and Relation Extraction (RE). Your task
is to ...<|end_of_text|><|start_header_id|>user<|end_header_id|>

Here is a text input: "Olmesartan medoxomil tablet ..." Here is the
list of input entities: ['Olmesartan medoxomil tablet',
...]...<|end_of_text|><|start_header_id|>assistant<|end_header_id|>

{"entities": [{"id": 0, "text": "Olmesartan medoxomil tablet", "type":
"Drug product"},...]}<|end_of_text|>
```

Prompt S1. Teacher model prompt and response. For brevity, we show a single in-context example. Special role tokens structure the conversation. The system prompt is shown in blue, the user input in orange, and the assistant output in red. The symbol "..." represents omitted parts of the prompt for readability. The full prompt is provided in the accompanying code repository.

S3. Benchmark datasets details and preprocessing

S3.1. NER benchmark datasets

We evaluate GLiNER-BioMed on eight biomedical NER datasets spanning a broad range of entity types. TAC (Roberts et al., 2017) consists of structured drug labels annotated with adverse drug event (ADE) mentions and contextual cues such as severity and negation. CADEC (Karimi et al., 2015) contains user forum posts related to medications and health issues, reflecting informal

language and covering ADEs, symptoms, and drug mentions. N2C2 2018 (Henry et al., 2020) focuses on medication-related concepts in discharge summaries, such as drug, dosage, frequency, route, duration, ADEs, and indications. BC5CDR (Li et al., 2016) is a widely used benchmark for recognizing chemical and disease mentions in PubMed abstracts. BioRED (Luo et al., 2022) broadens the biomedical NER scope to include genes, sequence variants, organisms, and cell lines in scientific abstracts. CHIA (Kury et al., 2020) covers clinical trial eligibility criteria, with annotations spanning conditions, procedures, devices, and other medical concepts. Biomed NER (Knowledgator Engineering, 2024) is a large-scale dataset covering a broad spectrum of biomedical, clinical, and regulatory entity types, including drugs, anatomical structures, phenotypes, and legal concepts. Finally, NCBI Disease (Doğan et al., 2014) is a curated dataset for disease name recognition in biomedical abstracts. An overview of total and test mention counts for each dataset, including the proportion of entities exceeding the model's maximum span length, is provided in Table S1. The label types and nomenclature are shown in Table S2.

Dataset	Total mentions	Test mentions	Test mentions exceeding 12 words (%)
BC5CDR	28 785	9809	0.00
Biomed NER	190 330	19 314	0.20
BioRED	20 419	3535	0.03
CADEC	8045	1302	0.23
CHIA	47 081	4745	1.64
N2C2	83 869	32 918	0.10
NCBI Disease	6892	960	0.21
TAC	28 122	13 478	0.04

Table S1. Overview of benchmark datasets used for evaluation. Mention counts refer to annotated entity spans. Test mentions refer to entities in the held-out test set. The last column reports the percentage of ground-truth entities in the test set that exceed the model's maximum span length of 12 words.

Dataset	Entity types
BC5CDR	Chemical, Disease
Biomed NER	Activity, Anatomical structure, Body substance, Cell or cell component, Chemical, Disorder, Drug, Event, Finding, Function, Gene or gene

	product, Geographical area, Group, Intellectual property, Language, Location, Medical procedure, Money, Organism, Organization, Person, Phenotype, Product, Regulation or law, Signaling molecule
BioRED	Cell line, Chemical entity, Disease or phenotype, Gene or gene product, Organism, Sequence variant
CADEC	Adverse drug event, Disease, Drug, Finding, Symptom
CHIA	Condition, Device, Drug, Measurement, Mood, Multiplier, Negation, Observation, Person, Procedure, Qualifier, Reference point, Scope, Temporal, Value, Visit
N2C2	Adverse drug event, Drug, Drug administration route, Drug dosage, Drug form, Drug strength, Frequency of drug administration, Reason for drug prescription, Treatment duration
NCBI Disease	Disease
TAC	Adverse drug event, Adverse drug event contextual modifier, Adverse drug event negation cue, Adverse drug event severity, Animal model, Drug class

Table S2. Overview of benchmark dataset entity labels, illustrating the specific nomenclature and casing used for GLiNER and LLM-based models.

S3.2. Preprocessing details

All texts were tokenized using spaCy, and entity annotations, originally provided in different formats such as BRAT, BIO tags, or XML, were mapped to token-level start and end indices. Where available, we retained official train/val/test splits; otherwise, we created stratified splits using fixed random seeds for reproducibility. Discontinuous entities were excluded to ensure compatibility with the GLiNER framework, and entity type names were standardized or renamed to ensure self-contained and interpretable label sets. To handle long documents, we chunked examples into segments capped at 512 subword tokens using the DeBERTa tokenizer, ensuring that no entity span was split across chunks.

S4. Small and base model

We developed base and small variants of GLiNER-BioMed, trained using the same procedure but with reduced parameter counts. As shown in Table S3, both GLiNER-BioMed-base and GLiNER-BioMed-small maintain strong zero-shot performance, outperforming all general-purpose baselines of similar size. At the base scale, the bi-encoder achieves a micro F1-score of 58.31%, exceeding its uni-encoder counterpart by 3.94 points ($p < 0.001$). At the small scale, the bi-encoder again leads with 56.93%, a 4.40-point improvement over the uni-encoder ($p < 0.001$). Remarkably, GLiNER-BioMed-small, despite having seven times fewer parameters than the large variant, achieves comparable performance to GLiNER-v2.5-large, with no statistically significant difference ($p > 0.05$).

Model	Micro F1	Macro mean F1	Macro median F1
**GLiNER-base**			
GLiNER-v1.0	41.61	24.98	10.27
GLiNER-v2.0	34.33	24.48	22.01
GLiNER-v2.1	40.25	25.26	14.41
GLiNER-news-v2.1	41.59	27.16	17.74
GLiNER-v2.5	46.49	30.93	25.26
GLiNER-BioMed	<u>54.37</u>	**36.20**	**41.61**
GLiNER-BioMed-bi	**58.31**	<u>35.22</u>	<u>32.39</u>
**GLiNER-small**			
GLiNER-v1.0	40.99	22.81	7.86
GLiNER-v2.0	33.55	21.12	15.76
GLiNER-v2.1	38.45	23.25	10.92
GLiNER-news-v2.1	39.15	24.96	14.48
GLiNER-v2.5	38.21	28.53	18.01
GLiNER-BioMed	<u>52.53</u>	**34.49**	**38.17**
GLiNER-BioMed-bi	**56.93**	<u>33.88</u>	<u>33.61</u>

Table S3. Zero-shot NER performance of GLiNER-BioMed base and small models compared with size-matched GLiNER baselines, aggregated over eight biomedical benchmarks. Bold: best within size; underlined: second-best.

REFERENCES

R. I. Doğan, R. Leaman, and Z. Lu. NCBI disease corpus: A resource for disease name recognition and concept normalization. Journal of Biomedical Informatics, 47:1-10, Feb. 2014. doi:10.1016/j.jbi.2013.12.006.

S. Henry et al. 2018 n2c2 shared task on adverse drug events and medication extraction in electronic health records. Journal of the American Medical Informatics Association, 27(1):3-12, Jan. 2020. doi:10.1093/jamia/ocz166.

S. Karimi et al. Cadec: A corpus of adverse drug event annotations. Journal of Biomedical Informatics, 55:73-81, June 2015. doi:10.1016/j.jbi.2015.03.010.

Knowledgator Engineering. [dataset] knowledgator/biomed_NER. Hugging Face, Sept. 2024. Available from: https://huggingface.co/datasets/knowledgator/biomed_NER.

F. Kury et al. Chia, a large annotated corpus of clinical trial eligibility criteria. Scientific Data, 7(1):281, Aug. 2020. doi:10.1038/s41597-020-00620-0.

J. Li et al. BioCreative V CDR task corpus: a resource for chemical disease relation extraction. Database, 2016:baw068, Jan. 2016. doi:10.1093/database/baw068.

L. Luo et al. BioRED: a rich biomedical relation extraction dataset. Briefings in Bioinformatics, 23(5):bbac282, Sept. 2022. doi:10.1093/bib/bbac282.

K. Roberts, D. Demner-Fushman, and J. M. Tonning. Overview of the TAC 2017 Adverse Reaction Extraction from Drug Labels Track. Text Analysis Conference (TAC) Workshop, 2017. Available from: https://tac.nist.gov/publications/2017/additional.papers/TAC2017.ADR_overview.proceedings.pdf.
